# Low-dose lymphocyte immunotherapy rebalances the peripheral blood Th1/Th2/Treg paradigm in patients with unexplained recurrent miscarriage

**DOI:** 10.1186/s12958-017-0315-9

**Published:** 2017-12-16

**Authors:** Mengyuan Liu, Xin Zhen, Hongyan Song, Junhao Chen, Xiaoling Sun, Xiaoqin Li, Jianjun Zhou, Guijun Yan, Lijun Ding, Haixiang Sun

**Affiliations:** 1 Department of Obstetrics and Gynecology, Center for Reproductive Medicine, the Affiliated Drum Tower Hospital of Nanjing University Medical School, Nanjing, China; 2 Department of Clinical Laboratory, the Affiliated Drum Tower Hospital of Nanjing University Medical School, Nanjing, China

**Keywords:** Recurrent miscarriage, Lymphocyte immunotherapy, Th1 cells, Th2 cells, Regulatory T cells

## Abstract

**Background:**

The published results regarding lymphocytes immunotherapy for unexplained recurrent miscarriage (uRM) patients are conflicting due to different screening criteria and therapeutic protocols. The objective of the present study is to evaluate the effectiveness of immunotherapy using low-dose lymphocytes in patients with uRM and Th1/Th2/Treg paradigm disorders.

**Methods:**

Sixty-four uRM patients who received low-dose lymphocytes immunotherapy served as the immunotherapy group, while the other 35 women who did not receive the treatment served as the control group. The proportions of peripheral blood Th1 cells, Th2 cells and Treg cells; and the concentration of TGF-β1 in serum were detected by flow cytometry and enzyme-linked immunosorbent assay (ELISA), respectively, before and after the immunotherapy.

**Results:**

The proportion of Th1 cells was significantly decreased while the proportions of Th2 cells and Treg cells were significantly increased in immunotherapy group patients after treatment. In addition, the concentration of TGF-β1 in serum was significantly higher after immunotherapy than before. Forty-three uRM patients achieved pregnancy after receiving immunotherapy and 5 patients underwent miscarriages in the immunotherapy group (11.6%, 5/43), while 8 of the 23 pregnant patients experienced a miscarriage in the control group (34.8%, 8/23; *p* < 0.05).

**Conclusions:**

Low-dose lymphocyte immunotherapy is beneficial for restoring balance in the Th1/Th2/Treg paradigm and improving pregnancy outcome in uRM patients.

**Trial registration:**

NCT03081325. ClinicalTrials.gov. Retrospectively registered July 2015.

**Electronic supplementary material:**

The online version of this article (10.1186/s12958-017-0315-9) contains supplementary material, which is available to authorized users.

## Background

Approximately 1–5% of all couples of reproductive age suffer from recurrent miscarriage **(**RM) [1]. This condition can lead to serious problems for patients’ health, spousal relationships, and quality of life. However, the etiology of RM is often unclear and may be multifactorial, with controversial diagnoses and treatment. Known etiologic causes include chromosomal anomalies, anatomical disorders, endocrine factors, thrombophilic factors, autoimmune abnormalities, and reproductive tract infections [2, 3]. However, approximately 50% of patients with RM do not manifest a clear cause, which is referred to as unexplained recurrent miscarriage (uRM), and appears to be mainly associated with alloimmune factors.

In a normal pregnancy, the mother accepts the fetus and maintains its development as an allograft that benefits from maternal-fetal immune tolerance. It has been reported in previous studies that the homeostasis of Th1/Th2 cytokines regulates maternal-fetal immune tolerance during pregnancy [4, 5]. A harmful role has been attributed to Th1 cells in pregnancy because some Th1-dependent effectors play important roles in acute allograft rejection, whereas the Th2-type cytokines seems to be central to the induction and maintenance of allograft tolerance [6]. Normal pregnancy is based upon Th2-type cellular immunity, while Th1 cellular immunity dominates the serum of uRM patients [7]. Furthermore, researchers have observed a shift in the peripheral Th1/Th2 ratio from Th1-dominant status to Th2-dominant status in successful pregnancies [8].

Maternal and fetal CD4^+^CD25^+^FOXP3^+^ regulatory T cells (Tregs) have been reported to contribute to the acquisition and maintenance of tolerance during pregnancy by suppressing maternal allogeneic immune responses. It has been reported that circulating CD4^+^CD25^+^ Treg increased during early pregnancy, which reached its peak during the second trimester and then declined in the postpartum period [9]. The percentages of CD4^+^CD25^+^ cells in peripheral blood were lower in uRM patients with early miscarriage compared with normal early pregnant women [10]. In addition, defects in the functions of Treg has also been documented in cases of uRM [11]. Low circulating CD4^+^ CD25^+^ Foxp3^+^ Treg cell levels can even predict miscarriage risk in newly pregnant women with a history of pregnancy loss [12]. Therefore, the Th1/Th2/Treg paradigm disorders may be a key target in uRM [13].

Immunotherapy with paternal lymphocytes has been used for uRM since the 1980s, and several groups have observed an increase in positive pregnancy outcomes after lymphocyte immunotherapy in uRM patients [14–18]. However, some researchers are still skeptical as to the efficacy of lymphocyte immunotherapy [19, 20].

The aim of this study was to investigate Th1/Th2/Treg paradigm disorders in the etiology of uRM, and to evaluate the efficacy of immunotherapy with low-dose of lymphocytes administered to uRM patients with Th1/Th2/Treg paradigm disorders.

## Methods

### Patients

All study patients were recruited from the Center for Reproductive Medicine, Affiliated Drum Tower Hospital of Nanjing University Medical School (Nanjing, China), between March of 2015 and July of 2016. Patients with uRM had 2 or more consecutive miscarriages (miscarriage was confirmed by ultrasound) before the 20th week of gestation and they all had one or more cellular immune disorder of the Th1/Th2/Treg paradigm prior to pregnancy including abnormally increased Th1 cells, abnormally decreased Th2 cells and abnormally decreased Treg cells [21]. Investigation of uRM patients was performed to exclude other possible causes of miscarriage including chromosomal anomalies, anatomical disorders (congenital uterine abnormalities, septate uterus, submucosal fibroids, intracavitary polyps, or cervical insufficiency), reproductive tract infections, endocrine factors (luteal phase deficiency, thyroid dysfunction, polycystic ovary syndrome or insulin resistance), autoimmune abnormalities (antiphospholipid syndrome, thyroglobulin antibodies, thyroid peroxidase antibodies) or thrombophilic status [1, 22, 23]. None of the couples was positive for infectious diseases (HIV, HBV, HCV, TPPA).

Sixty-four uRM patients who agreed to receive low-dose lymphocyte immunotherapy served as the immunotherapy group and signed informed consent forms before the treatment. The control group included 35 uRM patients who did not receive lymphocyte immunotherapy because of its uncertain effect. There was no significant difference between the 2 groups with respect to maternal age, number of miscarriages, or Th1/Th2/Treg paradigm (Table 1).

**Table 1:** Characteristics of uRM patients in the control and immunotherapy groups

	Control group	Immunotherapy group	*p* value
Total number	35	64	
Maternal age (*years*)	30.23 ± 4.98	30.89 ± 4.44	0.498
Number of miscarriages	2.23 ± 0.43	2.41 ± 0.68	0.166
Th1 cell proportion (*%*)	27.15 ± 9.00	30.02 ± 9.88	0.157
Th2 cell proportion (*%*)	0.96 ± 0.58	0.89 ± 0.39	0.478
Treg cell proportion (*%*)	2.16 ± 0.73	1.95 ± 0.74	0.174

This study received approval from the Nanjing Drum Tower Hospital Research Ethics Committee (IRB#SZ201–502), and the therapeutic protocol for this study has been registered in ClinicalTrials.gov (NCT03081325).

### Flow cytometric analysis

For the Th1 (CD3^+^CD4^+^IFN-γ^+^/CD4^+^) and Th2 (CD3^+^CD4^+^IL-4^+^/CD4^+^) cell proportion analyses, 50 μL peripheral venous blood from uRM patients were first incubated with a leukocyte activation cocktail and BD GolgiPlug (BD Biosciences, San Jose, CA, USA) for 5 h at 37 °C in a 5% CO_2_ humidified incubator. Then the cells were incubated with anti-human CD3 PerCP and anti-human CD8 APC antibodies (BD Biosciences, San Jose, CA, USA; see Additional file 1: Table S1. Antibody list for detailed antibody usage) away from light for 20 min. After surface staining, the cells were then fixed and permeabilized with FIX & PERM reagents (Life Technologies), and were stained with anti-human IFN-γ FITC and anti-human IL-4 PE antibodies (BD Biosciences, San Jose, CA, USA) for Th1 and Th2 detection. The normal range for the Th1 cell proportion was 9.0–31.0%, and the normal range for the Th2 cell proportion was 0.7–2.6%.

Treg (CD4^+^CD25^+^FOXP3^+^/CD4^+^) cells were detected using the Human Regulatory T Cell Staining Kit (eBioscience, Carlsbad, CA, USA). First, 50 μL peripheral venous blood from uRM patients were incubated with anti-human CD4 PerCP and anti-human CD25 FITC antibodies at room temperature (RT) for 30 min. Second, to stain for intracellular FOXP3, cells were fixed and permeabilized with FIX/PERM buffer from the kit and then stained with anti-human FOXP3 PE antibody. Samples were analyzed using a FACSCanto flow cytometer (BD Biosciences, Carlsbad, CA, USA). The normal range for the Treg cell proportion was 1.96–5.28%. All staining was performed according to manufacturer’s protocols. Isotype controls were used to discard non-specific background signal.

### Low-dose lymphocyte immunotherapy

About 15 ml of peripheral venous blood was drawn from patients’ husband in the immunotherapy group and PBMCs were isolated by Ficoll-Hypaque centrifugation (Tianjin Haoyang Biological Co. Ltd., China). After centrifugation, PBMCs were collected from the interphase layer and washed 3 times with sterile saline and resuspended in 0.7 ml of sterile saline at a concentration of 1.6 × 10^7^ cells/ml. The prepared PBMCs (1× 10^7^ cells) were administered 3 times intradermally every 3 weeks. The patients with uRM were then examined again with respect to the Th1/Th2/Treg paradigm to determine whether they conceived, and if not, they received another PBMCs intradermal administration. After conception, the uRM group of patients received lymphocyte immunotherapy twice every 8 weeks to strengthen their active immunity [24]. All uRM patients in the immunotherapy group and in the control group were followed up, after which they were allowed to prepare for conception. The follow-up considerations included gestation time and incidence of miscarriages, ectopic pregnancies, and pregnancy complications. uRM patients who had been pregnant for more than 20 weeks were defined as having a successful pregnancy.

### Enzyme-linked immunosorbent assay

Serum was collected from blood samples from uRM patients in the immunotherapy group before and after the lymphocyte immunotherapy. The concentration of TGF-β1 in the serum was detected with cytokine-specific ELISA kits following the manufacturer’s instructions (Boster Bio, China).

### RNA isolation and quantitative real-time PCR

Total RNA was isolated from PBMCs from uRM patients in the immunotherapy group using TRIzol reagent (Invitrogen, Carlsbad, CA, USA) according to the manufacturer’s instructions. cDNA was synthesized using a PrimeScript RT reagent kit (Bio-Rad Laboratories, Hercules, CA, USA). The mRNA-specific oligonucleotide primers used for qRT-PCR analysis were as follows:

IFN-γ, forward 5′- TGCAGAGCCAAATTGTCTCC -3′ and reverse 5′- TGCTTTGCGTTGGACATTCA-3′;

IL-4, forward 5′- TTTGCTGCCTCCAAGAACAC -3′ and reverse 5′- GTCGAGCCGTTTCAGGAATC -3′;

FOXP3, forward 5′- GTGGCCCGGATGTGAGAAG -3′ and reverse 5′- GGAGCCCTTGTCGGATGATG -3′;

and human 18S, forward 5′- CGGCTACCACATCCAAGGAA -3′ and reverse 5′- CTGGAATTACCGCGGCT -3′. Each qRT-PCR reaction comprised the following components: 2 μL cDNA, 2 μL ddH_2_O, 5 μL SYBR Green PCR Master Mix (Bio-Rad Laboratories, Hercules, CA, USA), and 0.5 μL each of the forward and reverse primers. The qRT-PCR was performed on a MyiQ Single Color Real-Time PCR Detection System (Bio-Rad Laboratories, Hercules, CA, USA) using the following procedure: 95 °C for 3 min; 94 °C for 10 s; 60 °C for 30 s; and 72 °C for 30 s. The fold change in expression of each gene was calculated using the 2^-△△CT^ method, with 18S rRNA used as an internal control.

### Statistical analyses

Data are presented as means ± SD. The maternal age, number of miscarriages, and Th1/Th2/treg paradigm between the immunotherapy and control groups were evaluated using the independent *t* test. The effects of immunotherapy on the proportion of Th1 cells, Th2 cells, and Treg cells in the immunotherapy group before and after immunotherapy were evaluated by paired *t* test. Fisher’s exact probability test was used to detect the differences in miscarriage rates and live birth rate between the groups. A *P*-value <0.05 was considered to be statistically significant. Statistical analyes were performed using GraphPad Prism 5 software.

## Results

### Low-dose lymphocyte immunotherapy exerted an obvious treatment effect on Th1/Th2/Treg paradigm disorder in uRM patients

Of the 64 uRM patients in the immunotherapy group, Th1 cells were abnormally increased in 33 of 64 (51.56%, Table 2), Th2 cells were abnormally decreased in 14 of 64 (21.88%), and Treg cells were abnormally decreased in 39 of 64 patients (60.94%).

**Table 2:** The proportion of Th1 cells, Th2 cells, and Treg cells in uRM patients in the immunotherapy group before and after lymphocyte immunotherapy

	Before immunotherapy	After immunotherapy	*p* value
Total Th1	32.67 ± 9.05	31.38 ± 9.18	0.075
Total Th2	0.89 ± 0.40	1.00 ± 0.46	0.142
Total Treg	1.78 ± 0.64	2.33 ± 0.64	<0.001
Abnormal Th1 (*n = 33*)	37.69 ± 5.33	35.83 ± 6.86	0.038
Abnormal Th2 (*n = 14*)	0.44 ± 0.25	0.84 ± 0.42	0.013
Abnormal Treg (*n = 39*)	1.53 ± 0.30	2.27 ± 0.63	<0.001

**Fig. 1:**
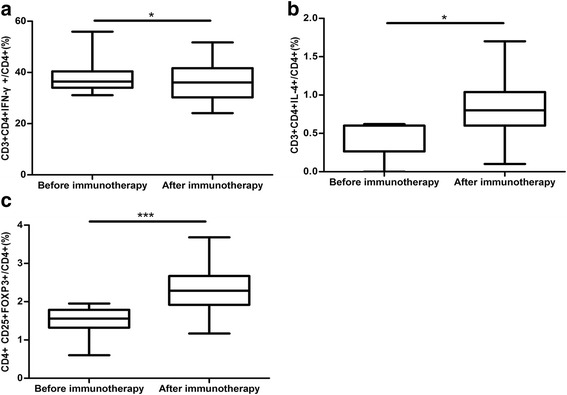
The treatment effect of low-dose lymphocyte immunotherapy on Th1/Th2/Treg paradigm disorder. *,*P* < 0.05;**,*P* < 0.01;***,<0.001

The proportion of abnormal Th1 cells in uRM patients was significantly decreased after immunotherapy (37.69 ± 5.33% vs. 35.83 ± 6.86%, respectively; *n* = 33, *P* = 0.038; Fig. 1a and Table 2). The proportion of abnormal Th2 cells was significantly increased after immunotherapy (0.44 ± 0.25% vs. 0.84 ± 0.43%, respectively; *n* = 14, *P* = 0.013; Fig. 1b and Table 2); and the proportion of abnormal Treg cells was also significantly increased after immunotherapy (1.53 ± 0.30% vs. 2.27 ± 0.63%, respectively; *n* = 39, *P* < 0.001; Fig. 1c and Table 2).

### TGF-β1 concentrations and mRNA expression for IFN-γ, IL-4, and FOXP3 after immunotherapy

**Fig. 2:**
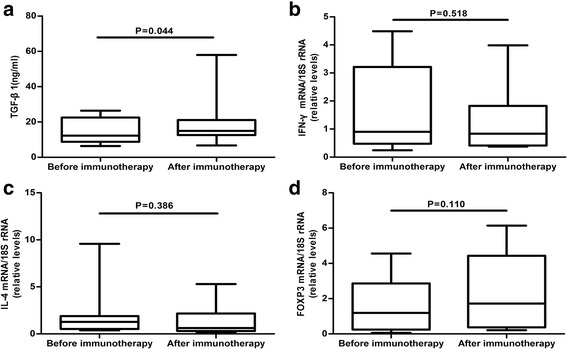
TGF-β1 concentrations and mRNA expression for IFN-γ, IL-4, and FOXP3 of uRM patients before and after low-dose lymphocyte immunotherapy

As shown in Fig. 2a, the concentration of TGF-β1 cytokines in the serum of uRM patients in the immunotherapy group was significantly increased after immunotherapy vs. controls (14.76 ± 7.34 ng/ml vs. 19.55 ± 12.50 ng/ml, respectively; *n* = 22, *P* = 0.044). However, we did not find significant difference in the mRNA expression level of IFN-γ, IL-4 and FOXP3 before and after immunotherapy (*P* = 0.518, 0.386, 0.110, respectively; Fig. 2b-d).

### Pregnancy outcome in uRM patients following low-dose lymphocyte immunotherapy

Currently, 23 of 35 uRM patients in the control group became pregnant and 15 of the pregnant patient had delivered healthy babies (60.87%, Table 3), one patient was pregnant for more than 20 weeks, but 8 of the 23 pregnant patients had miscarriages (34.78%).

**Table 3:** Pregnancy outcome in uRM patients with or without lymphocyte immunotherapy

	Control Group	Immunotherapy Group	*p* value
Pregnancy	23(23/35)	43(43/64)	
Delivery	14(60.87%)	32(74.42%)	0.068
≥20 weeks	1(4.35%)	5(11.63%)	–
Exfetation	0(0.00%)	1(2.32%)	–
Abortion	8(34.78%)	5(11.63%)	0.048

Within the immunotherapy group, 43 of the 64 uRM patients had achieved pregnancy. Of these, 32 patients delivered healthy babies (74.42%, Table 3), the live birth rate of patients showed a tendency to increase after low-dose lymphocyte immunotherapy (60.87% vs. 74.42%, *P* = 0.068); 5 patients were pregnant for more than 20 weeks (11.63%), according to epidemiologic studies, 80% of miscarriage occurs before 12 weeks of pregnancy, while less than 8% of pregnancy losses occur after 20 weeks [3]. By 20 weeks of gestation, the fetus remains in a relative safe state and the pregnancy is defined as successful pregnancy. And one patient experienced an ectopic pregnancy. The remaining 5 patients experienced repeated miscarriages in the first trimester (11.63%), the miscarriage rate was significantly lower than the uRM patients in the control group (11.63% vs. 34.78%, *P* < 0.05). We did not observe any serious side effects during the immunotherapy process except the reactions at the application site of concentrated paternal lymphocytes.

## Discussion

Immunotherapy with lymphocytes is one of the most extensively studied treatment methods for patients with uRM. However, the published results regarding this treatment are conflicting [24]. In the present study, we observed improved pregnancy outcome in patients with unexplained recurrent miscarriage after low-dose lymphocyte immunotherapy, with increased live birth rate and decreased miscarriage rate for patients in the immunotherapy group than that in the control group.

Although our study followed a protocol similar to that of other studies, some improvements were made in our therapeutic protocol. First, we used appropriately chosen screening criteria for selecting patients who were to undergo lymphocyte immunotherapy [25]. Immunotherapy offered a significantly higher success rate in patients with immune abnormalities compared to patients without any such abnormalities. Many studies also showed that immune imbalance among Th1, Th2, and Treg cells is involved in the immune pathology of uRM [16, 26]. This suggests that modulation of immune imbalance in the Th1/Th2/Treg paradigm can be a therapeutic option for uRM patients. In the present study, we enrolled uRM patients who had immune abnormalities such as an increased proportion of Th1 cells or a decreased proportion of Th2 cells or Treg cells, and used the Th1/Th2/Treg paradigm as a criterion for therapeutic effect.

Second, the dose of lymphocytes administered to uRM patients was very important in immunotherapy. It is reported that immunotherapy performed with high-dose lymphocytes (classified as more than 1 × 10^8^ lymphocytes) exerted only a slight effect on improving the live birth rate in patients with uRM, while immunotherapy performed with low-dose lymphocytes (classified as less than 1 × 10^8^ lymphocytes) improved the success rate significantly for patients with uRM [21]. In our study, we used 1 × 10^7^ cells per application, intending to properly rebalance the peripheral blood Th1/Th2/Treg paradigm.

Third, some investigators have reported that using immunotherapy with stored fresh and not refrigerated lymphocytes decreased miscarriage rates and improved the live birth rate per embryo transfer, and lymphocyte immunotherapy performed before and during pregnancy produced a better outcome compared with that performed only before pregnancy [24, 27]. Additionally, the effects caused by immunotherapy were best observed when the lymphocytes were administered intradermally, as compared to subcutaneous injection [21]. Based on these studies, we performed the lymphocyte immunotherapy with fresh, non-stored blood before and during pregnancy, and applied it intradermally to uRM patients.

Several immunologic mechanisms have been associated with uRM. Most studies indicate higher levels of activated immune cells and associated cytokines, and lower levels of regulatory cells and associated cytokines in patients with uRM; and the Th1/Th2/Treg paradigm appears to play an important role in maintaining immune homeostasis between the fetal and maternal units [25]. Th1-type cells secrete IL-2 and IFN-γ, which enhance the immune cytotoxicity of NK cells, thus inhibiting embryonic implantation, trophoblast growth, and embryonic development. Th2-type cells secrete IL-4 and IL-10 to protect the embryo from attack by the immune system by inhibiting the Th1-type immune response [28]. Treg cells suppress immune responses to a broad range of non-microbial and microbial antigens, and indirectly limit the immune inflammation that inflicts tissue damage by employing multiple mechanisms of suppression [29]. Treg cells are therefore a key player within the regulation of maternal immune tolerance; and lower numbers and altered function of Treg cells have been associated with adverse pregnancy outcomes in both human and animal studies [30].

All uRM patients enrolled in this study had at least one disorder in their Th1/Th2/Treg paradigm, and we hypothesized that this might be the etiology of uRM. Our results showed that low-dose lymphocyte immunotherapy could effectively decrease the abnormally high levels of Th1 cells and increase the abnormally low levels of Th2 and Treg cells. We also found that the concentration of TGF-β1 in serum was significantly increased after immunotherapy. TGF-β1 is known to promote FOXP3 expression by inducing Treg differentiation from CD4^+^CD25^+^ T cells and inhibit the activity of many cytokines, including IFN-γ and tumor necrosis factor-alpha (TNF-α). This can then lead to a restoration in the balance of the Th1/Th2/Treg paradigm [31]. In our study, this rebalance was beneficial to pregnancy outcome in the immunotherapy group.

## Conclusions

Our study suggests that Th1/Th2/Treg paradigm disorders constitute one etiology of uRM, and that low-dose lymphocyte immunotherapy has an obvious effect on the disorder and improves the outcome of pregnancy.

## Additional file

Additional file 1: Table S1. Antibody list. (DOC 34 kb)

## Abbreviations

ELISA: Enzyme-linked immunosorbent assay
FOXP3: Forkhead box P3
IFN-γ: Interferon γ
IL-4: Interleukin 4
PBMCs: Peripheral blood mononuclear cells
RM: Recurrent miscarriage
TGF-β: Transforming growth factor-β
Th1: T helper 1 cells
Th2: T helper 2 cells
TNF-α: Tumor necrosis factor-alpha
Tregs: Regulatory T cells
uRM: unexplained recurrent miscarriage
