# Supplementary material for: Low-dose lymphocyte immunotherapy rebalances the peripheral blood Th1/Th2/Treg paradigm in patients with unexplained recurrent miscarriage
Source: Reprod Biol Endocrinol. 2017 Dec 16;15:95. doi: 10.1186/s12958-017-0315-9 (PMC5732480; doi:10.1186/s12958-017-0315-9)
Supplement: Additional file 1: Table S1. — Antibody list. (DOC 34 kb) [file 12958_2017_315_MOESM1_ESM.docx]

**Table S1.** Antibody list

| **Antibody name** | **Source** | **Catalog # Clonality** | **Species of origin** | **Concentrations** |
| --- | --- | --- | --- | --- |
| CD3 PerCP | BD Bioscience, San Jose, CA, USA | 347344 Monoclonal | Mouse | 1:2.5 |
| CD8 APC | BD Bioscience, San Jose, CA, USA | 340584 Monoclonal | Mouse | 1:10 |
| Anti-Human  IFN-γ FITC | BD Bioscience, San Jose, CA, USA | 340449 Monoclonal | Mouse | 1:2.5 |
| Anti-Human  IL-4 PE | BD Bioscience, San Jose, CA, USA | 340451 Monoclonal | Mouse | 1:2.5 |
| Anti-Human  CD4 FITC | eBioscience, Carlsbad, CA, USA | 22-0425 Monoclonal | Mouse | 1:2.5 |
| Anti-Human  CD25 APC | eBioscience, Carlsbad, CA, USA | 22-0425 Monoclonal | Mouse | 1:2.5 |
| Anti-Human  FOXP3 PE | eBioscience, Carlsbad, CA, USA | 12-4776 Monoclonal | Mouse | 1:2.5 |
